# Intracellular bacteria in cancer—prospects and debates

**DOI:** 10.1038/s41522-023-00446-9

**Published:** 2023-10-09

**Authors:** Lena Schorr, Marius Mathies, Eran Elinav, Jens Puschhof

**Affiliations:** 1 Microbiome and Cancer Division, German Cancer Research Center, Heidelberg, Germany; 2 Faculty of Biosciences, Heidelberg University, Heidelberg, Germany; 3 Systems Immunology Department, Weizmann Institute of Science, Rehovot, 7610001 Israel

**Keywords:** Microbiome, Cellular microbiology

## Abstract

Recent evidence suggests that some human cancers may harbor low-biomass microbial ecosystems, spanning bacteria, viruses, and fungi. Bacteria, the most-studied kingdom in this context, are suggested by these studies to localize within cancer cells, immune cells and other tumor microenvironment cell types, where they are postulated to impact multiple cancer-related functions. Herein, we provide an overview of intratumoral bacteria, while focusing on intracellular bacteria, their suggested molecular activities, communication networks, host invasion and evasion strategies, and long-term colonization capacity. We highlight how the integration of sequencing-based and spatial techniques may enable the recognition of bacterial tumor niches. We discuss pitfalls, debates and challenges in decisively proving the existence and function of intratumoral microbes, while reaching a mechanistic elucidation of their impacts on tumor behavior and treatment responses. Together, a causative understanding of possible roles played by intracellular bacteria in cancer may enable their future utilization in diagnosis, patient stratification, and treatment.

## Introduction

The prospect of bacteria potentially impacting cancer gained traction in the late 19th century, with reports such as that of William Russell and colleagues suggesting that microorganisms may reside within tumors^1,2^. After a period of early excitement and attempts to identify a unified bacterial cause of cancer^3^, reproducible research on carcinogenic bacteria proved elusive for almost a century. Consequently, the bacterial theory of cancer was abandoned, and the fields of microbiology and oncology independently evolved for several decades with little intersection. In cancer research, genomic aberrations and deregulated signaling pathways have, for long, taken center stage amongst other modulating factors, as key drivers of tumorigenesis^4^. Conversely, vastly improved culturing and identification methods have revealed early reported “cancer microbes” to be likely contaminants^5^. While groundbreaking discoveries on *Helicobacter pylori* (*H. pylori*) in stomach ulcers and consequent gastric neoplasms transformed our view of microbes in cancer^6^, it is only in the past 15 years that the prospect of cancer microbiome research has been revisited on a larger scale^7^. Technological developments in next-generation sequencing (NGS) that enabled the characterization of microbiome-rich samples, such as fecal, vaginal and oral microbiomes, have revealed distinct stool microbial signatures associated with different stages of colorectal cancer (CRC)^8–11^ and possibly other cancers^7,12^.

Beyond changes in the stool microbiome, analyses of tumor sequencing data recently suggested that specific microbes may reside within tumors and their microenvironment^13–16^. Interestingly, some of these cancer-associated low-biomass bacteria were suggested to reside inside tumor and immune cells. Ample evidence attests to the enrichment of intracellular bacteria, such as *Fusobacterium nucleatum* (*F. nucleatum*) in CRC^16–18^. Generalization of such findings to other tumors that had been previously considered sterile is suggested by some studies^14,15^ and debated by others^19,20^, therefore meriting future confirmatory studies.

Intracellular pathogens have been extensively studied in infectious diseases. For many bacterial pathogens, intracellular localization constitutes an important, at times obligatory, component of their lifestyle. Intracellular localization bestows numerous advantages on the invading microbe, ranging from immune escape to a favorable nutritional environment and a platform for replication and dissemination. In contrast to infectious pathogens, interactions and target cells of cancer-specific intracellular bacteria remain understudied. Addressing the critical question of whether low-biomass microbial inhabitants are consistently present in tumors, and whether they bear functional implications on cancer development, progression, and therapy response is faced by multiple technical and conceptual challenges. Nevertheless, it is already yielding insightful discoveries. For example, certain bacteria detected in tumors, such as genotoxic *Escherichia coli (E. coli*) and enterotoxigenic *Bacteroides fragilis* (*B. fragilis*) have been linked to the production of metabolites that promote inflammation and DNA damage, contributing to the initiation and growth of tumors^21–23^. Moreover, some bacteria may influence the response of tumors to chemotherapy and immunotherapy^24,25^. Some of these effects may be explained by bacteria metabolizing and inactivating chemotherapeutic drugs^26^. Others can alter an immune response in the context of immune checkpoint blockade in modulating the response to immunotherapy^27,28^.

**Fig. 1:**
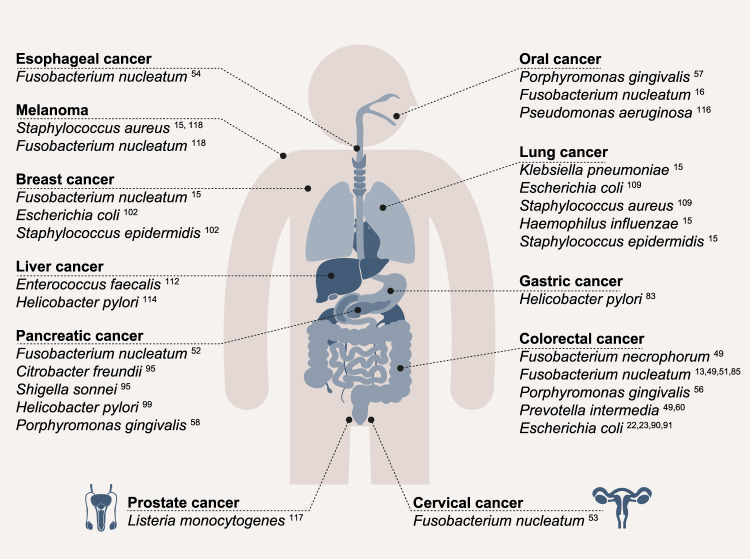
Tumor-associated bacterial species suggested to occur intracellularly. An overview of bacterial species that are suggested to be enriched in various cancers while exhibiting the capacity to invade host cells.

While more than a century has passed since the first reports suggesting that microorganisms may exist within cancer cells, the notion of intracellular bacteria in cancer is being revisited. As microbiology and oncology come to intersect anew, we aim to describe in this review the challenges facing the field and highlight where lessons may be learned from decades of microbiology research in a non-cancer context. Here, we provide an overview of intratumoral bacteria research, focusing on intracellular bacteria that have been suggested to colonize different tumor types and cancer models (Fig. 1). The number of studies focusing specifically on intracellular bacteria in cancer remains limited, and proper separation between intratumoral and intracellular effects is often lacking. For this reason, in this review, we refer to “intratumoral” microbes as tumor-associated microorganisms whose precise localization is unclear, while denoting them “intracellular” if they are suggested to reside within cells of the tumor microenvironment (TME). The broader relationships of the bacterial gut microbiome^7,14,27,29^ and other microbial kingdoms^30,31^ with cancer are reviewed elsewhere.

## Lifestyles of intracellular bacteria

Intracellular pathogens have been studied for decades and yielded key insights into the mechanisms driving bacterial invasion, induction of host damage and evasion of host defense. Concepts gained from these studies, summarized below, could prove useful to the study of similar bacterial behaviors and host impacts in the cancer setting.

### Strategies of bacterial invasion

Pathogen interaction with host receptors drives their adherence to host cells and triggers actin cytoskeleton rearrangements, leading to cellular invasion. Intracellular bacteria may reside in isolation, or in mixed consortia, depending on the species^32,33^. Bacteria that are classified as intracellular occur inside the host cell for at least parts of their lifecycle. Obligate intracellular bacteria, such as *Rickettsia spp*. and *Chlamydia spp*., have an exclusively intracellular lifestyle, while facultative intracellular bacteria, such as *Salmonella spp*. or *Listeria monocytogenes* (*L. monocytogenes*), can also proliferate extracellularly under certain conditions. A pathogen’s path into the host cell fundamentally varies between active invasion and passive phagocytosis, of which only active invasion will be discussed here. Bacteria can enter the host cell actively via so-called zipper or trigger mechanisms (Box 1)^34^. In some cases, actin polymerization and depolymerization lead to the formation of membrane protrusions, which ultimately result in the uptake of bacteria within a vacuole. Target cells of invasion are diverse and include among others epithelial cells, endothelial cells, keratinocytes, and different types of immune cells such as macrophages^34–39^. Intracellular localization provides bacteria with several advantages, such as protection from circulating immune cells and nutrient availability^40^.

Box 1: Overview of the trigger and zipper mechanism **Trigger and Zipper mechanism**

Intracellular bacteria invade non-phagocytic host cells via the trigger or zipper mechanism.

**Zipper**: The bacterium enters the cell in a receptor-mediated, zipper-like manner. Surface invasins and adhesins of the bacterial membrane interact with receptors on the host membrane. This interaction triggers signaling cascades that lead to reorganization of the host cytoskeleton by actin polymerization. This results in the formation of membrane protrusions that enclose and internalize the bacterium.

**Trigger**: The bacterium inserts effector molecules into the host cell, where they lead to actin rearrangement and then ultimately membrane protrusions, allowing the bacterium to enter the host. The effector molecule application typically occurs through type III (T3SS) and type IV (T4SS) secretion systems.

### Host bacterial sensing

To counter pathogenic bacterial engagement and associated cellular and tissue damage, the host has developed sophisticated systems to recognize and combat intracellular bacteria. Recognition by the innate immune system of the host is mostly mediated by pattern-recognition receptors (PRRs) such as surface-bound Toll-like receptors (TLRs) or cytosolic NOD-like receptors (NLRs) and retinoic acid-inducible gene I (RIG-I) receptors, among others. These enable constant surveillance of different compartments of the cell for signs of infection. Activation of PRRs by invading bacteria triggers a variety of inflammatory cascades, including nuclear factor kappa B (NF-κB) activation, inflammasome formation, and cytokine release, coupled with induction of an inflammation-induced infected cell death termed pyroptosis, which collectively elicit broad rejection responses to bacterial invasion^41,42^. In addition, upon bacterial sensing, microRNAs (miRNA) play a role in regulating various downstream processes such as cell cycle, autophagy or immune responses^43^ and hence can favor or combat infections. The adaptive immune system is also able to sense intracellular bacteria by host presentation of bacteria-derived peptide fragments on major histocompatibility complexes (MHC), sometimes challenged by bacterial antigenic variation^44^. Interaction of MHC peptide complexes with effector T cells leads to activation and proliferation of these cells, which in turn initiates differentiation and activation of other immune cells, ultimately triggering a multi-channeled antimicrobial immune response^45^.

### Survival and proliferation strategies

To counter these defense mechanisms in a constant “arms race”, intracellular bacteria have evolved mechanisms of adaptation and resistance^41^. After entering the host by either the trigger or zipper mechanism, the bacteria end up in a phagosome. Phagosomes normally fuse with lysosomes to phagolysosomes, which digest their contents. Bacteria have devised intricate mechanisms of blocking this fusion, thereby escaping the phagosome, as well as other means of neutralizing bacteriolytic function, while surviving inside the host cell. For example, *Mycobacterium tuberculosis* (*M. tuberculosis*) uses an interplay of an impenetrable cell envelope, detoxification, and radicals and acquires the Ras-related protein (Rab-5A), which blocks fusion with the lysosome in enabling survival in the phagosome^45^. Listeria, Rickettsia, and Shigella escape from the vacuole and replicate in the cytosol^46^ through movement facilitated by hijacking of the host cytoskeleton via actin polymerization. Additionally, intracellular bacteria, such as Rickettsia, Burkholderia, *L. monocytogenes*, and *Shigella flexneri* (*S. flexneri*), can exploit the actin cytoskeleton for spreading between host cells. These pathogens utilize the host cell actin network to form protrusions into adjacent cells, leading to a double-membrane vacuole forming in the new host cell, composed of the membrane of the previous and the new host^47^. Other bacteria may inhibit autophagy, a degradation and recycling process that is effective against invading bacteria^48^. Similar invasion, evasion, and survival mechanisms may be used by intracellular bacteria in the cancer setting, and merit future studies.

## Composition and localization of intratumoral bacteria

Some tumors, and in particular CRC, are convincingly shown to harbor intratumoral and even intracellular bacteria. Indeed, most CRC-associated microbes, such as *F. nucleatum*^15–18,49–55^, *P. gingivalis*^56–59^, and *Prevotella intermedia* (*P. intermedia*)^49,60,61^ are suggested to reside intracellularly. Other, traditionally considered “sterile” tumors are also suggested to feature low-biomass bacterial communities, as shown by several genomic-based^14,15,20,62^, and imaging-based approaches^15,16^. Of note, some of the genomic approaches utilized by these studies have been recently challenged^19,63^, with a definite resolution of these debates, and high-resolution characterization of intratumoral bacteria meriting future studies.

NGS, ranging from characterization of the 16S rDNA gene to global characterization of the bacterial pan-genome using shotgun metagenomic sequencing, is extensively utilized in unraveling fecal cancer-associated microbiome signatures at low host DNA contamination levels^8,9^. In contrast, efforts to profile the low-biomass tumor-resident microbiome prove to be more challenging, given a substantial excess of human reads, imperfections in reference databases, experimental and computational contaminations, and severe batch effects caused by varying collection methods, sample processing, and sequencing pipelines^14,62,64^. Suggested solutions include novel low-biomass sequencing approaches, in which five regions of the 16S rRNA gene are simultaneously amplified and sequenced, enabling higher coverage and resolution^15,65^. These amplicons can then be computationally combined using Short MUltiple Regions Framework (SMURF)^65^, a platform built specifically for this method. Of note, 5 region 16S rRNA sequencing is highly sensitive and bacterial DNA can be found in many sources of contamination, making it challenging to distinguish between low-biomass bacterial signals originating from the tissue and those originating from environmental contamination^66,67^. Such distinction requires careful assessment by multiple controls to bioinformatically filter out interfering signals^15^. Additional efforts to improve disentangling of true low-biomass signals from contamination and noise focus on improved harmonization of experimental and computational pipelines^68^, the use of multiple technical and biological controls, and validation of sequencing-based results with additional, non-genomic modalities. With these modalities providing some solution to the low-biomass microbial challenges, future development is needed to further disentangle true microbial signals from noise.

Metatranscriptomic analysis can provide further functional insights into microbial consortia and their relationships with their host^69^. Dual RNA sequencing approaches have emerged in 2012^32^ to gain insights into simultaneous gene expression of host cells, invading bacteria and their interactions^32^. These modalities have evolved to enable the assessment of co-infections of different bacterial species, and even viruses and bacteria, in decoding inter-species and inter-kingdom interactions^70^. Single-cell RNA sequencing may further minimize bulk contamination by cellular and genetic heterogeneity and reveal the extent to which individual cells are targeted and shaped by invading bacteria. For example, Galeano Niño et al. established a method called invasion–adhesion-directed expression sequencing (INVADEseq), focusing on cell attachment and invasion with spatial resolution. In INVADEseq, primers targeting a conserved region of the bacterial 16S rRNA locus enable the generation of cDNA libraries containing bacterial transcripts from human cells associated with bacteria. While good resolution may be achieved, distinction between invaded and cell-adjacent bacteria still remains a major obstacle^16^. Using this method in oral squamous cell carcinoma (OSCC), Fusobacterium and Treponema colonization were associated with macrophages and aneuploid epithelial cells^16,71^. Another tool, Single-cell Analysis of Host–Microbiome Interactions (SAHMI)^52^, relies on recovering and denoising microbial signals from single-cell sequencing data of the host tissue and mapping it to a microbial reference genome. In addition, computational prediction tools such as Host–Microbe Interaction PREDictor (HMI-PRED), used for prediction of protein–protein interactions, may enable further elucidation of host–microbe interactions^72^.

Imaging techniques are increasingly used to complement genomic low-biomass microbial signals^15^. These include immunohistochemistry (IHC), FISH and high-resolution electron microscopy. The recently introduced correlative focused ion beam/scanning electron microscopy (c-FIB/SEM) combines volume electron microscopy and fluorescence microscopy, thereby enabling better understanding of host-microbe interactions at the 3D ultrastructural level^73^. Utilization of fluorochrome-conjugated bacteria-specific antibodies coupled with permeabilization of host samples may enable intracellular microbial imaging^18,74^. However, killing of both bacteria and host cells limits the use of this approach to endpoint assays. Stable labeling of intracellular bacteria, such as *F. nucleatum*, is challenged by difficulties in genetic modification, despite recent progress^75^. Bacterial chemical labeling^50,76^, while beneficial for short-term analyses, suffers from attenuation of the signal by serial dilution, promoted by cell division over time. Fluorescently labeled d-alanine may offer a solution to this challenge, as it is metabolically incorporated into the bacterial cell wall and thus may enable the detection of living, metabolically active tumor-associated bacteria upon incubation with fresh tumor samples^77^.

Recent work from the Bullman group incorporated several state-of-the-art spatial profiling methods in better characterizing bacterial niches in tumors^16^. RNAscope-fluorescence in situ hybridization (RNAscope-FISH) enabled the visualization of bacterial RNA within individual host cells, thereby revealing locations of bacteria within tumors. Using the digital spatial profiling platform GeoMX, expression of 77 proteins related to anti-tumor immunity was correlated with tumor characteristics. Through application of 10X Visium spatial transcriptomics, positional information could also be documented in tumor niches. Using these methods, it was suggested that most intratumoral bacteria are located in micro-niches at the tumor margin, featuring an immunosuppressive and poorly vascularized microenvironment^16^.

### Suggested intracellular bacteria across tumor and tissue types

Studies utilizing the above technologies suggest that tumors may harbor unique microbial signatures. The most extensive evidence demonstrating such tumor-residing bacteria and their tumor-modulatory function involves gastric and colorectal cancer. A definite level of proof regarding presence, extent, and possible functions of intratumoral microbiomes, and the prospect of their utilization in cancer diagnosis^14^, remains debated^19^ and awaits future validation.

#### Gastric cancer

*H. pylori* was the first, and currently only bacterium recognized by the World Health Organization as a carcinogen to date. *H. pylori* constitutes a prototypical driver of gastritis and subsequently gastric cancer via diverse mechanisms such as induction of chronic inflammation^78–80^ and modulation of wingless-related integration site (Wnt) signaling^81^, amongst others. The many direct and indirect contributions of *H. pylori* to gastric cancer development have been reviewed elsewhere^82^. Of interest to intratumoral microbiome research, some of the effects conferred by *H. pylori* have been suggested to be linked to attachment and potential invasion of gastric epithelial cells^74,75^, supported by the presence of *H. pylori* in gastric cancer tissue^83^.

#### Colorectal cancer

Colorectal cancer is the third most common cancer worldwide and the second leading cause of cancer deaths in the US^84^. Due to its proximity to a rich luminal microbiome, it is unsurprising that it has been studied extensively for intratumoral and intracellular bacteria. As noted above, among the CRC-associated bacteria with intracellular localization, *F. nucleatum* is the best studied species to date. Several studies have detected an enrichment of *F. nucleatum*, in human adenomas and CRC compared to healthy colon tissue samples^13,51,85^. The invasive behavior of patient-derived *F. nucleatum* subspecies was validated by functional assays in CRC cell lines^49,74,86^. Intracellular *F. nucleatum* is also enriched in inflammatory bowel disease (IBD), a condition predisposing to CRC, especially in patients suffering from an active disease compared to those in remission^87^. *F. nucleatum* strains from patients featured an invasive behavior in 2D cell line assays, which correlated with IBD status^74^. *Fusobacterium necrophorum* (*F. necrophorum*), another member of the Fusobacterium genus with an invasive behavior^88^, has also been associated with CRC^49^. Another periodontal-linked bacterium, *P. gingivalis*, was enriched in CRC compared to healthy adjacent tissue^56^ and suggested to reside intracellularly^16^ while featuring an invasive behavior^89^. *P. gingivalis* dominantly occurs in the oral cavity, where it resides in gingival epithelial cells and is linked to periodontitis and OSCC^57,89^. Comparing paired adenocarcinoma and polyp samples, a higher abundance of *P. intermedia* was detected in adenocarcinoma samples^60^, and it was suggested to reside, at least partly, intracellularly^16,49,61^. Pathogenic strains of *E. coli* are also recurrently found in CRC^21–23,90^. Some of those are able to thrive within macrophages^91^ and could be cultured from tumor tissue after gentamycin treatment^92^, suggesting that they may feature a capacity to intracellularly survive. Of note, a recent report suggests a close interplay between attachment and genotoxicity for some of these strains^92^.

#### Pancreatic cancer

Pancreatic ductal adenocarcinoma (PDAC) constitutes the most common form of pancreatic cancer, accounting for more than 85% of all cases^93^. In PDAC, Campylobacter, Leptotrichia, *F. nucleatum* and *Clostridioides difficile* were suggested as intratumoral species based on single-cell sequencing data^52^. Further studies detected *Citrobacter freundii* and *Shigella sonnei*^94^ as part of the pancreatic cancer TME, while other studies suggested that these bacteria occur intracellularly^95,96^. A meta-analysis from 2011 concluded that *H. pylori*, which was shown to be capable of invading host cells^97^, enhances the risk of pancreatic cancer^98^. *P. gingivalis* has also been reported to be dominantly enriched in patients with pancreatic adenomas and possibly correlated with PDAC disease progression^58^. PDAC was also suggested to bear fungi^15,99,100^, but some of these findings were recently contested^63^, meriting future research.

#### Other cancer types

*F. nucleatum*^15^, *Staphylococcus epidermidis* (*S. epidermidis*), *E. coli*^101^, and other members of the genus Staphylococcus, Streptococcus, and Lactobacillus^102^ were suggested to be present in human **breast cancer** samples. The abundance and potential intracellular localization of microbes in breast cancer awaits definite resolution. *F. nucleatum* was suggested to be enriched in **cervical cancer**^53^ and **esophageal cancer**^54^ compared to adjacent healthy tissue. Associations and potential functional roles for intracellular *Chlamydia spp*. have been described in **cervical** and **ovarian cancer**^103–105^. This pathogen has been shown to decrease p53 signaling and DNA damage response^106^, in facilitating the pathogen’s intracellular replication, induction of ROS production and DNA damage^107^. Analysis of a TCGA lung adenocarcinoma dataset, a subtype of **lung cancer**, suggested that *E. coli* and *Staphylococcus aureus* (*S. aureus*) may reside within these tumors^108^. *S. epidermidis, Haemophilus influenzae* (*H. influenza*), and *Klebsiella pneumoniae* (*K. pneumoniae*)^15^, were suggested to reside in lung cancer samples in separate studies^109^. Using transmission electron microscopy, intracellular bacteria could be visualized in intrahepatic cholangiocarcinoma, a form of **liver cancer**, and surrounding tissue, but no further characterization of the bacteria was performed^110^. A presence of *Enterococcus faecalis (E. faecalis*) was demonstrated in hepatocellular carcinoma samples and suggested to have a prominent role in liver carcinogenesis^111^, also in the in vivo setting^112^. In hepatocellular carcinoma, the presence of *H. pylori* was detected only in cancer samples, while it was absent in healthy controls^113^. Based on spatial analysis, Galeano Niño et al. revealed that the genera Parvimonas, Peptoniphilus and Fusobacterium were most abundant in an **oral cancer** subtype, OSCC^16^. *Pseudomonas aeruginosa*, which is capable of an intracellular lifestyle^114^, was detected in the same cancer subtype^115^. *L. monocytogenes* was detected in the **prostate cancer** TME^116^, while *S. aureus* was suggested to be present in **melanoma**^15,117^. It is noteworthy that in these studies, low-biomass approaches utilized different protocols, isolation methods and sample handling techniques, which can collectively lead to variations and inconsistencies between studies. For example, *B. fragilis* was suggested to be more abundant in CRC in one study^118^, while other studies reported no significant differences in its abundance^119^, which could stem from differences in participant characteristics and varying experimental designs. Given recent debates, these low-biomass findings merit future validation.

### Synergies between intracellular bacteria

Many microbiome-modulated diseases are not driven by single pathogens, but rather by synergistic consortia that can evolve into a dysbiotic state that impairs host homeostasis via concerted activities^120^. For example, gingival *P. gingivalis* infection in germ-free mice induces periodontal disease only in the presence of commensal communities, through joint promotion of polymicrobial biofilms via regulation of cytokine levels^121^. Similar concepts could also be relevant in the cancer context. Initial studies show that *F. nucleatum*-positive CRC tissues featured non-random co-colonization with commensals such as *B. fragilis* or *P. intermedia*, whereas tissues lacking *F. nucleatum* featured different bacterial colonization patterns^49^. These co-colonization patterns may bear functional importance. For example, communities containing both *F. nucleatum* and *P. gingivalis* featured a higher rate of invasion of gingival epithelial cells as compared to those featuring either of these species without the other^122^. Further investigation is needed to determine the extent to which bacterial composition and co-occurrence patterns causally shape their modulatory activities impacting cancer.

### Routes of bacterial colonization into tumors

Routes by which intratumoral bacteria may reach and persist in the TME remain elusive to date. It is conceivable that bacteria from the adjacent normal tissue become enriched at tumor sites during tumorigenesis due to the changed microenvironment and easier tissue access upon disruption of epithelial and mucus barriers, for example, the gastrointestinal tract. For many other tumor types and even most gastrointestinal tumors, additional routes are plausible, including transmission of bacteria to distant sites via the bloodstream or the lymphatic system^123,124^.

Oral cavity-to-gut translocation of bacteria constitutes an active field of investigation in CRC and other cancer research. Bacteria of the oral cavity are involved in local inflammatory diseases such as periodontal disease and are also enriched in various, seemingly unrelated, tumor tissues. Intriguingly, a 10-year study found periodontal patients to have an increased risk of cancer development, particularly pancreatic cancer^125^, but also other cancers such as lung cancer, head and neck cancer, abdominal and esophageal cancer, breast cancer, and CRC^126^. Several periodontal bacteria, such as *F. nucleatum* and *P. gingivalis*, feature an invasion and colonization capacity even in their gingival tissue of origin^127,128^. Elucidation of effector similarities of such oral microbiome bacteria upon migration into tumor sites merits future mechanistic research. *F. nucleatum*, a well-studied invasive cancer-related bacterium, is believed to migrate from its natural habitat within the oral cavity^124^ via the bloodstream. From the bloodstream, local enrichment in colorectal adenocarcinoma is suggested to be mediated by binding between bacterial fibroblast activation protein-2 (Fap2) and host epithelial d-galactose-β(1-3)-N-acetyl-d-galactosamine (Gal-GalNAc), which is overrepresented at CRC sites^129^. In addition, *F. nucleatum* can bind to the salivary protein statherin via FomA, which functions in biofilm formation and is known to bind especially to *P. gingivalis*^51,130^. In PDAC, gut bacteria can migrate from the anatomically connected upper gastrointestinal tract into the tumor^131^. Of note, translocation of gut microbes to sites like the liver can precede tumor formation^111^. After entering the TME, factors such as nutrient-rich niches, low pH, necrotic foci, hypoxia, or abundant blood supply may support bacterial colonization^132–134^. Similar considerations may apply to microbial colonization of metastases. In a murine breast cancer model, bacteria detected in circulating tumor cells were also enriched in lung metastasis sites, suggesting that some bacteria, and specifically intracellular bacteria, may migrate to metastatic sites within tumor cells via the systemic circulation^102^. Further evidence suggests that certain strains of *E. coli* can disrupt the gut vascular barrier and support CRC cells at multiple stages of their metastatic dissemination^135^.

## Potential impacts of intracellular bacteria on cancer phenotypes

The above observations, suggestive of the occurrence of tumor-associated bacteria, constitute an intriguing starting point of investigation of potential causation, e.g., whether tumor-residing or even intracellular bacteria may impact tumorigenesis, cancer progression, and treatment responsiveness. This “chicken-and-egg” dilemma constitutes a formidable challenge that requires sophisticated experimental models and techniques to detect causes and mechanisms.

### Bacterial invasion models

**Fig. 2:**
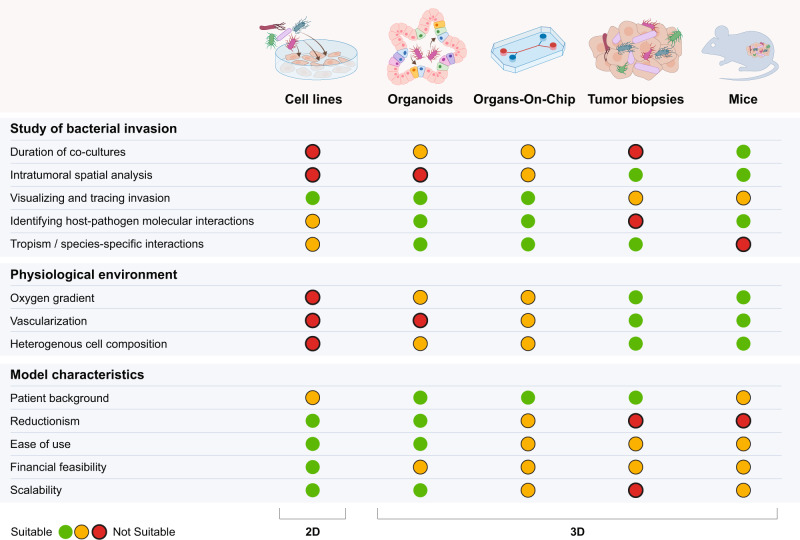
Experimental models and approaches. Key features of cell-based models with varying complexity focusing on the study of bacterial invasion, the physiological environment, and model characteristics. Advantages and disadvantages for multiple parameters are compared and evaluated by a traffic light system.

To mechanistically study bacterial invasion, in vitro models of varying complexity are often combined with antibiotic protection assays that use non-penetrant antibiotics, such as gentamicin, to enrich viable intracellular microbes^86,136–141^. There are different approaches and models to study invasion that vary in their ease of use and the representation of in vivo physiology, a summary of which is depicted in Fig. 2. One of the most commonly used invasion assays is based on the co-incubation of 2D cell lines, such as Caco-2 cells derived from CRC, with microbes that actively invade the host cells^18,25,50,74,86,138,139^. Such 2D studies revealed important interactions between surface components of bacteria and the host, invasion, and the induction of oncogenic and inflammatory pathways upon infection^18,50,86,129^. For example, the use of such platforms uncovered the importance of the lectin Fap2, expressed on *F. nucleatum*, binding to Gal-GalNAc on host cells. This interaction, in turn, drives an accumulation of Fusobacteria in CRC^50,129^, upregulation of inflammatory markers upon invasion^13,18,50^, and direct modulation of the TME^138^. Moreover, *F. nucleatum* is suggested to enhance CRC progression through its FadA adhesin, which attaches to epithelial cadherin (E-cadherin) and may activate the Wnt/β-catenin signaling pathway^86^ leading to enhanced proliferation^86^. Such 2D invasion assays have also proved invaluable in testing means to interfere with bacterial tumor cell invasion. For example, galactosides were discovered to interfere with Fap2-mediated invasion of *F. nucleatum* into CRC cells^50^. However, many proposed cancer-associated bacteria thrive in anaerobic conditions, making it difficult to mimic a suitable 2D environment that would phenocopy their in vivo niche. In addition, 2D in vitro invasion models remain limited to timeframes of a few hours, which may be too short for the effects of viable intracellular bacterial invasion and related metabolic crosstalk to become apparent^142–144^, while the immortalized character of most cell lines makes it difficult to elucidate differences between healthy and cancer cells.

To overcome these limitations, new models enable more physiologically relevant conditions for such invasion studies, which are summarized in Fig. 2. Organoids represent a useful model to study pathogen-host interactions because they mimic the polarized epithelial cell layer enclosing a luminal compartment^138,139^. There are various ways to use organoids as co-culture platforms, including microinjection of bacteria into the hypoxic lumen from where they can actively invade the surrounding epithelial layer in the right orientation. Early steps of invasion of bacteria such as *Salmonella enterica* serovar Typhimurium (*S. Typhimurium*) were decoded using such platforms. Further usage of invasion platforms, including spheroids^145^, and transwell invasion assays^146^ is likely to reveal additional functional impacts of intracellular bacteria in cancer.

Organs-On-Chip (OOC) models, like CRC OOCs, enable the study of bacterial tumor invasion at an enhanced level of complexity, by replicating environmental features, including stretch, flow, and even complex gut microbial compositions^76,147,148^. Using Caco-2 colonized OOCs^149^, factors such as crypt-like structures, peristalsis, and flow were shown to have a substantial impact on Shigella invasion. Mouse models can further elucidate the impacts of intracellular and intratumoral bacteria on tumor growth in a complete host environment. As one example, *F. nucleatum*-positive colorectal tumors were subcutaneously transplanted into immunodeficient mice and tracked over time. Using this model, viable *F. nucleatum* could be maintained over time, while antibiotic administration reduced tumor growth^49^. In the C57BL/6 Apc^min/+^ CRC mouse model, increased tumor formation occurred upon gavage of *F. nucleatum*, while demonstrating a key role of FadA in its tumor-promoting activity^140^. Enhanced tumorigenesis could be likewise demonstrated upon transplantation of the *P. gingivalis* pre-infected pancreatic cancer cell line PANC1, suggested to be mediated by enhancement of phosphatidylinositol 3-kinase (PI3K)/protein kinase B (Akt) signaling^128^.

### Modulation of immune responses

**Fig. 3:**
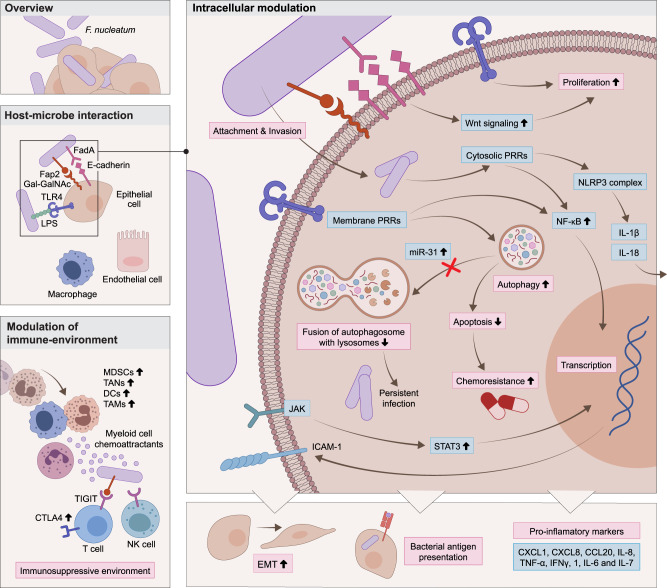
Modulation of host cells by *F. nucleatum*. The opportunistic pathogen *F. nucleatum* impacts host cell behavior via numerous extra- and intracellular mechanisms. *F. nucleatum* interacts with the host via protein interactions such as Fap2-Gal-GalNAc or FadA-E-cadherin and can invade cell types such as epithelial cells, endothelial cells or macrophages. Intracellular *F. nucleatum* is sensed by membrane-bound and cytosolic PRRs, mediating NF-κB activation and expression of inflammatory molecules such as precursors of IL-1b and IL-18 that are activated by cleavage by inflammasomes and then released from the host cell. Activation of cytosolic PRRs also affects increased expression of ICAM-1. The binding of LPS to TLR4 causes upregulation of autophagy, linked to chemoresistance. *F. nucleatum* also leads to the upregulation of miR-31 which inhibits the fusion of autophagosomes with the lysosome and enables persistent infection. Consequences of *F. nucleatum* invasion include increased JAK-STAT signaling, enhanced secretion of proinflammatory markers, EMT phenotype and bacterial antigen presentation on HLA molecules. The binding of FadA to E-cadherin influences downstream Wnt signaling, leading to enhanced proliferation. Extracellularly, *F. nucleatum* induces secretion of myeloid chemoattractants that recruit TANs, DCs and TAMs, leading to suppressed activity of CD4^**+**^ T cells. Direct interaction of *F. nucleatum* with immune cells via Fap2-TIGIT binding, expressed on T and NK cells, mediates decreased cytotoxicity and ultimately inhibited killing activity. CCL20 C-C Motif Chemokine Ligand 20, CTLA4 cytotoxic T-lymphocyte associated protein 4, CXCL C-X-C motif chemokine ligand, DCs dendritic cells, E-cadherin epithelial cadherin, Fap2 fibroblast activation protein-2, *F. nucleatum* *Fusobacterium nucleatum*, Gal-GalNAc d-galactose-β(1-3)-N-acetyl-d-galactosamine, IFN interferon, ICAM-1 intercellular adhesion molecule 1, IL interleukin, JAK Janus kinase, LPS lipopolysaccharide, miRNA-31 microRNA-31, NF-κB nuclear factor kappa-light-chain-enhancer of activated B cells, NK natural killer, NLRP3 NLR family pyrin domain containing 3, PRRs pattern-recognition receptors, STAT signal transducer and activator of transcription, TAMs tumor-associated macrophages, TANs tumor-associated neutrophils, TIGIT T-cell immunoreceptor with Ig and ITIM domains, TLRs Toll-like receptors, TNF tumor necrosis factor, Wnt wingless-related integration site.

Infection of host cells often generates potent immune responses. For example, endothelial cells invaded by the obligate intracellular bacterium *Orientia tsutsugamushi* elicit an interferon response^69^ promoting immune cell recruitment^150^. *S. Typhimurium* that infects macrophages can replicate intracellularly, resist host reactive oxygen species (ROS) responses and actively induce macrophage polarization^150^. Within the cytosol, *F. nucleatum* can be sensed via PRRs such as RIG-I^151^ or alpha kinase 1 (ALPK1), leading to NF-κB activation. This leads to various signaling cascades such as expression of the proinflammatory cytokines interleukin (IL)-6, IL-8, and tumor necrosis factor alpha (TNF-α), upregulation of adhesion molecules^152^, enhanced proliferation or autophagy^51,151^. Suggested mechanisms of intra- and extracellular F. nucleatum affecting proliferation, autophagy and TME modulation of the host are depicted in Fig. 3. Autophagy is modulated by various intracellular bacteria, affecting their niche and enabling intracellular survival. *F. nucleatum*, for example, may promote initiation of autophagy through downregulation of miR-18a* and miR-4802^43^, while autophagosome fusion with the lysosome may be inhibited via intracellular *F. nucleatum*-mediated upregulation of miR-31^48,153^. An expansion of myeloid-derived immune cells including tumor-associated macrophages (TAMs), tumor-associated neutrophils (TANs) and myeloid-derived suppressor cells (MDSCs) in a *F. nucleatum*-inoculated CRC mouse model was accompanied by T-cell suppression and increased expression of immunosuppressive molecules such as CTLA4 and arginase-1^17,154^. Recruitment of myeloid cells by intratumoral bacteria may also contribute to inflammation via activation of the Janus kinase/signal transducer and activator of transcription (JAK-STAT) pathway and secretion of interleukins and chemokines^87^. Modulation of natural killer (NK) cells by bacteria in the TME can further promote an immunosuppressive environment. For example, interaction of Fap2, expressed by *F. nucleatum*, with the human inhibitory receptor T-cell immunoreceptor with Ig and ITIM domains (TIGIT) expressed on immune cells such as NK cells and T cells, abrogated NK cell-mediated killing of human tumor cells^138^. Similarly, *F. nucleatum*-induced Fap2-dependent mechanisms can drive lymphocyte apoptosis^155,156^. Tumor regions featuring a high bacterial load may correlate with reduced vascularization, thereby contributing to the formation of an immunosuppressive environment^16^. Intratumoral bacteria may also modulate the TME adaptive immune response via the presentation of bacteria-derived peptide fragments on host antigen-presenting cell HLA complexes. Indeed, in melanoma, peptides of species such as *F. nucleatum* and *S. aureus* were presented on TME antigen-presenting cells. Some of these peptides appear to be potentially immunogenic, thereby bearing a capacity to activate adaptive immune responses while promoting tumor infiltration of lymphocytes and generating an inflammatory immune response^117^. Similar findings are emerging in glioblastoma multiforme^157^, where multiple bacteria-derived antigens were suggested to be recognized by T cells of the TME. However, several of the suggested microbe-harboring tumors appear to be cold, or relatively immunotolerant. This apparent lack of tumor antibacterial immune reactivity, despite the suggested presence of highly immunogenic bacteria, merits future studies.

### Impact on tumor metastasis

**Fig. 4:**
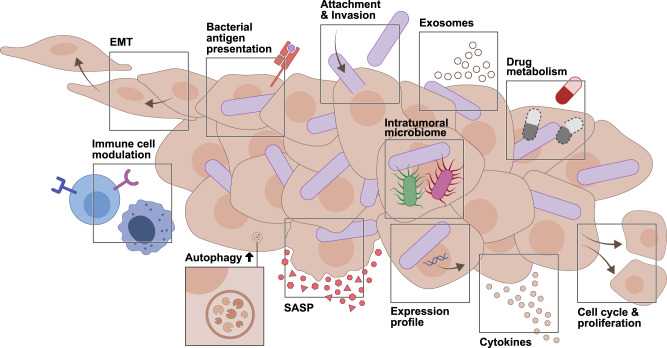
Cancer-related processes possibly influenced by intracellular bacteria. Bacteria colonizing host tumor cells may alter a multitude of cancer-related features. These include gene expression changes triggered by host bacterial sensing, SASP or alterations in proliferation, and induction of metastasis-linked behavior such as migration, invasion, EMT or release of exosomes. Intracellular bacteria may modulate autophagy, immune cell recognition and antigen presentation, potentially impacting cancer immune recognition. Ultimately, bacteria-driven host modulations can lead to alterations in drug metabolism and consequently therapy response, thereby impacting the outcome of cancer therapies. EMT epithelial–mesenchymal transition, SASP senescence-associated secretory phenotype.

Intratumoral and intracellular microbes may also regulate the metastatic cascade. Whole genome sequencing revealed a similarity of 99.9% between *F. nucleatum* isolates from primary CRC and liver metastasis, indicating likely bacterial migratory characteristics between these tumor sites^49^. Microbial regulation of the metastatic process may involve a variety of mechanisms. Epithelial–mesenchymal transition (EMT) is a dynamic process of epithelial conversion to a mesenchymal phenotype, characterized by a gradual loss of epithelial features such as epithelial cell adhesion molecule (EpCAM) expression or strong cell-cell contacts^158^. *F. nucleatum* may induce transcriptional features of EMT such as upregulation of Vimentin, Snail or Slug^16,159,160^. Higher intratumoral *F. nucleatum* abundance is associated with an increase in ALPK1, which in turn results in upregulation of intercellular adhesion molecule 1 (ICAM-1) on the host cell surface. This leads to increased attachment of CRC cells to endothelial cells which is suggested to promote EMT and ultimately metastasis^152^. Other suggested microbial mechanisms impacting host tumor cell metastatic potential include the secretion of bioactive products, as exemplified by *F. nucleatum*-derived short-chain fatty acid formate that was recently linked to CRC cell stemness and invasion^161^. In addition, *F. nucleatum* may induce the expression of pro-migratory and pro-metastatic genes in a CRC cell line, impact the motility of cancer cells, and modulate the virulence factor Fap2-driven host cell binding and suppress T-cell infiltration^16^. Keratin7 (KRT7) is reported to be upregulated in tumors upon *F. nucleatum* presence and was suggested to enhance lung metastasis in a murine CRC model^162^. In addition, tumor-associated bacteria may modulate cell-cycle signaling pathways^16^ and induce senescence in the host. Unlike apoptotic cells, senescent cells, which undergo irreversible cell-cycle arrest, remain viable. The senescence-associated secretory phenotype (SASP) involves the release of factors, which promote inflammation and malignancy (Fig. 4). In CRC, invasive *P. gingivalis* is enriched and correlated with higher levels of butyrate, possibly inducing SASP^59^. Regarding the secretion profile, *F. nucleatum* invasion also induced increased exosome secretion of CRC cells carrying metastasis-related miRNAs and proteins, which, in turn, were internalized by uninfected cells and activated Wnt/β-catenin signaling, enhancing migration and proliferation^163^.

### Effects on drug metabolism and treatment responsiveness

In addition to their ability to disrupt endogenous cellular processes, bacteria can metabolize drugs, thereby potentially affecting tumor responses. Several studies suggest that bacteria-mediated drug metabolism may induce drug activation or promote adverse effects. For example, gut bacteria-derived beta-glucuronidase can metabolize the chemotherapy irinotecan in a manner promoting increased mucosal toxicity^164^. In a larger in vitro screen in which 30 FDA-approved drugs were co-incubated with bacteria, 10 compounds were found to feature a decreased efficacy, while 6 featured an increased efficacy^165^. Geller et al. reported that Gammaproteobacteria may confer gemcitabine resistance to CRC cell lines by metabolizing the drug to its inactive form by the bacterial enzyme cytidine deaminase. Consequently, antibiotic treatment promoted a regained responsiveness to gemcitabine in a CRC mouse model. Importantly in this study, the most common species in human PDAC belonged to the Gammaproteobacteria class, while 14 out of 15 PDAC-derived bacterial cultures mediated gemcitabine resistance upon co-culture with CRC cell lines. Recently, *E. coli*-mediated inactivation of the nucleoside analog 5-fluorouracil (5-FU) was associated with a poorer therapy response. Interestingly, 5-FU was also suggested to inhibit the growth of *F. nucleatum*^166^, which might further contribute to its therapeutic activity. Neoadjuvant chemoradiotherapy with fluoropyrimidines (which are activated to 5-FU) decreased the prevalence of *F. nucleatum* from 58 to 26% in locally advanced rectal cancers, while the persistence of *F. nucleatum* following treatment predicted a relapse^167^. Co-cultivation of 5-FU and Oxaliplatin with *F. nucleatum* suggested that an altered chemotherapeutic drug response was linked to a decrease in two miRNAs (miR-4802 and miR-18a*) driving enhanced expression of autophagy-related proteins^25^. Understanding the effects of microbial colonization in tumors on treatment effectiveness and adverse effects may enable tailoring treatment to the individual based on their microbial profiles, in optimizing dosing while minimizing adverse effects.

### Targeting intracellular bacteria in cancer treatment

Intratumoral bacteria may represent potential therapeutic targets in cancer treatment. For example, antibiotic treatment of mice transplanted with *F. nucleatum*-positive patient-derived CRC xenografts reduces tumor size and cancer cell proliferation, indicating that bacterial suppression may support tumor growth suppression^49^. However, targeting intratumoral pathogens remains challenging, as in many cases internalized antibiotics do not reach the minimum inhibitory concentration in the TME^168^, while high doses are associated with adverse effects on the host and its microbiome. Currently tested intracellularly acting antimicrobials include sulfonamides, quinolones, tetracyclines, and beta-lactams^169^. However, emerging antibiotic resistance requires the development of further therapeutic strategies that overcome low cellular permeability and microbial resistance. Explored modalities include intracellularly acting antimicrobial peptides (AMPs), such as fungal plectasin, which suppresses intracellular methicillin-resistant *S. aureus* (MRSA). Nonetheless, AMP activity may be abrogated upon reaching the host cell cytoplasm. Cell-penetrating peptides (CPP) can translocate to the nucleus, mostly via endocytic pathways, where they may exert antibacterial effects. CPP can also be used as translocation backbones to direct drugs into infected cells. As an example, the CPP Tat was coupled to the antibiotic gentamicin, leading to a reduction of intracellular *E. coli* K1^170^. Singh et al. proposed to conjugate the antibiotics polymyxin B sulfate and sushi peptide to gold nanoparticles, in effectively targeting intracellular *Salmonella typhi*^171^. Utilization of bacteriophages represents another experimental approach in specifically eliminating tumor-associated bacteria. For example, phages targeting *F. nucleatum*, combined with irinotecan were able to reduce tumor growth, while selectively inhibiting CRC-associated bacterium *F. nucleatum* in vivo^172^. Similarly, phages directed against adherent invasive *E. coli* reduced tumor burden in a CRC mouse model^173^. These pioneering efforts may be further optimized by the use of phage combinations suppressive target bacterial strains through different receptors, as was recently shown in the context of IBD^174^. However, phage treatment of intracellular bacteria is challenged by limited access into host cells, a changed environment altering intracellular phage-bacterial engagement, and the necessity for phages to transit between host cells in targeting multiple intracellular bacteria.

### Utilization of intracellular bacteria in cancer treatment

Intracellular bacteria may be potentially utilized as medical interventions or treatment delivery vectors. For example, Salmonella used as a nonpathogenic delivery system targeting therapeutic proteins into the cytoplasm induced a reduced tumor growth and metastasis in mouse models^175^. Modification of the *Photorhabdus asymbiotica* type VI secretion system, which plays a role in macrophage invasion and intracellular survival, enabled the delivery of custom payloads into human cells^176–178^. Additionally, intratumoral bacterial peptide human leukocyte antigens (HLA) presentation on host cells may enable the harnessing of antibacterial immunity in cancer immunotherapy. As the bacterial antigens are recognized as non-self, they could serve as targets for immunotherapy stimulating an immune response^117^. In 2004, an attenuated *S. Typhimurium* systemically administered to a mouse model of melanoma featured a sustained ability to invade tumor cells. Prior vaccination of mice that generated Salmonella-specific T cells resulted in a substantial improvement in tumor load. Tumor cell invasion was suggested to play a role in this effect, as the use of non-invasive *S. Typhimurium* resulted in a marked attenuation of the anti-tumor response^179^.

## Limitations and challenges in the study of intracellular tumor-residing bacteria

Despite recent advances in our collective understanding of the possible presence and activities of microbes in cancer, the field remains in its infancy and faces major challenges and obstacles. While the intracellular presence of Fusobacterium is well documented^15–18,49–55^, the presence of microbial communities, consisting of bacteria^14,15,62^ and fungi^14,15,99^ within different tumors that were commonly considered sterile has been recently debated^19,63,180^ and merits definite characterization by future studies. Such low-biomass tumor microbiome characterization is challenged by a variety of technical and biological challenges and potential biases stemming from contaminations, batch effects, erroneous read allocation, and imperfection in analytical pipelines, while lacking resolution to identify intracellular localization of bacteria in conjunction with tumor phenotype measurements. Examples of the challenges facing such research are presented in Box 2. The study of bacterial invasion using animal models and organoids will further contribute to our understanding of the true extent of intratumoral bacterial colonization and its potential functions. In such future experiments, the importance of addressing experimental and computational contaminations through the use of robust and diverse controls and analytical tools cannot be overstated and is highlighted by the long-standing debate as to the existence of a placental microbiome which has only recently been resolved (as likely not being present) by a large consortium effort^181^.

Disentangling the effects of extracellular microbes from those of true tumor cell-penetrating intracellular bacteria may enable to quantify their true impact. Importantly, recent advances have highlighted the likely heterogeneous distribution of bacteria between different specimens and even within the same sample^16^. Despite advances in bacterial identification, variations on a species and subspecies level are often not easy to decode, especially in the low-biomass microbial setting, with different strains often grouped solely by their genus, disregarding their diversity and presence of virulence factors in specific strains.

An additional major challenge stems from the crucial need to transform our level of understanding from correlation, association, and prediction to experimental determination of causation and molecular mechanisms. Even for some of the most commonly reported intracellular bacteria, like *F. nucleatum* in CRC, conflicting reports suggest that some strains lack stable gut colonization, coupled with a lack of CRC-promoting effects^182,183^. Careful evaluation of bacterial engraftment, invasion and downstream impacts on the invaded host, assessed across multiple experimental platforms, may be necessary to draw robust conclusions on an intratumoral or even intracellular microbial causative roles in cancer. Beyond single-species bacterial studies, elucidation of bacterial-bacterial interactions, as well as trans-kingdom interactions with viruses^184–186^, fungi^30,187,188^ and eukaryotic microorganisms will constitute fascinating topics of research in the coming decade. Progression from the use of cellular systems and animal models to clinical translation will be complex but presents opportunities to harness the gained knowledge in developing novel diagnostics and therapeutics.

In conclusion, some studies suggest that seemingly sterile tumors may harbor a unique low-biomass microbiome, whose bacterial constituents may partially reside within host tumor and immune cells. Future advances in imaging, experimental models, sequencing and data analysis may validate these findings, while accounting for confounders and possible contaminations. Even such definite proofs will only form the basis for the exploration of many unknowns. The rules of engagement by which bacteria populate tumors in specific intratumoral niches remain to be determined. Causal temporal and spatial relations between bacteria and their invaded TME host cells remain elusive, and their downstream effects on tumorigenesis and treatment response are only beginning to be unraveled. The coming decade will likely see a deepening research effort exploring these exciting topics, while exploiting the gained knowledge towards a putative development of novel cancer interventions.

Box 2: Challenges and open questions facing the prospect of intracellular bacteria in cancer ***Technical challenges:***

1. …robustly characterize the presence, abundance, and composition of intratumoral microbiomes in diverse cancer types.
2. …account for environmental contaminants, batch effects, read misalignment, and reference database imperfections.
3. …resolve the presence of genomically identified taxa that are unlikely to colonize the human body.
4. …elucidate the precise (intracellular) localization of specific members of the intratumoral microbiome.
5. …integrate in vivo and next-generation in vitro models to accurately reflect human bacteria-tumor interactions.
6. …achieve single-cell resolution of bacterial invasion effects.
7. …distinguish between intracellular and extracellular effects.

***Biological questions:***

1. Do bacterial signals identified within tumor cells represent intact, live organisms?
2. How stable is bacterial localization within tumor cells?
3. Is bacterial tumor colonization random or pre-determined?
4. Which bacteria actively invade cancer and TME immune cells, and which are passively ingested by tumor-associated phagocytes?
5. Can intratumoral bacteria causally impact cancer and its treatment?
6. How do bacterial strains differ in their invasion and tumorigenic capacity?
7. Which microbe–environment and microbe–microbe interactions matter in the context of invasion and cancer biology?

### Reporting summary

Further information on research design is available in the Nature Research Reporting Summary linked to this article.

### Supplementary information

Reporting Summary

## References

[1] Russell W (1890). An address on a characteristic organism of cancer. Br. Med. J..

[2] Butlin HT (1884). Malignant tumours and parasitism. Br. Med. J..

[3] Glover T (1926). Progress in cancer research. Canada Lancet and Practitioner.

[4] Hanahan D, Weinberg RA (2011). Hallmarks of cancer: the next generation. Cell.

[5] ACS. Unproven methods in cancer management: Livingston-Wheeler therapy. *CA Cancer J. Clin.* **40**, 103–108 (1990).

[6] Polk DB, Peek RM (2010). *Helicobacter pylori*: gastric cancer and beyond. Nat. Rev. Cancer.

[7] Cullin N, Azevedo Antunes C, Straussman R, Stein-Thoeringer CK, Elinav E (2021). Microbiome and cancer. Cancer Cell.

[8] Thomas AM (2019). Metagenomic analysis of colorectal cancer datasets identifies cross-cohort microbial diagnostic signatures and a link with choline degradation. Nat. Med..

[9] Wirbel J (2019). Meta-analysis of fecal metagenomes reveals global microbial signatures that are specific for colorectal cancer. Nat. Med..

[10] Zeller G (2014). Potential of fecal microbiota for early-stage detection of colorectal cancer. Mol. Syst. Biol..

[11] Yachida S (2019). Metagenomic and metabolomic analyses reveal distinct stage-specific phenotypes of the gut microbiota in colorectal cancer. Nat. Med..

[12] Kartal E (2022). A faecal microbiota signature with high specificity for pancreatic cancer. Gut.

[13] Kostic AD (2012). Genomic analysis identifies association of Fusobacterium with colorectal carcinoma. Genome Res..

[14] Poore GD (2020). Microbiome analyses of blood and tissues suggest cancer diagnostic approach. Nature.

[15] Nejman D (2020). The human tumor microbiome is composed of tumor type-specific intracellular bacteria. Science.

[16] Galeano Nino JL (2022). Effect of the intratumoral microbiota on spatial and cellular heterogeneity in cancer. Nature.

[17] Kostic AD (2013). Fusobacterium nucleatum potentiates intestinal tumorigenesis and modulates the tumor-immune microenvironment. Cell Host Microbe.

[18] Castellarin M (2012). Fusobacterium nucleatum infection is prevalent in human colorectal carcinoma. Genome Res..

[19] Gihawi A, Cooper CS, Brewer DS (2023). Caution regarding the specificities of pan-cancer microbial structure. Microb. Genom..

[20] Sepich-Poore, G. D. et al. Reply to: Caution regarding the specificities of pan-cancer microbial structure Preprint at *bioRxiv*10.1101/2023.02.10.528049 (2023).

[21] Nougayrede JP (2006). *Escherichia coli* induces DNA double-strand breaks in eukaryotic cells. Science.

[22] Arthur JC (2012). Intestinal inflammation targets cancer-inducing activity of the microbiota. Science.

[23] Dejea CM (2018). Patients with familial adenomatous polyposis harbor colonic biofilms containing tumorigenic bacteria. Science.

[24] Gharaibeh RZ, Jobin C (2019). Microbiota and cancer immunotherapy: in search of microbial signals. Gut.

[25] Yu T (2017). *Fusobacterium nucleatum* promotes chemoresistance to colorectal cancer by modulating autophagy. Cell.

[26] Geller LT (2017). Potential role of intratumor bacteria in mediating tumor resistance to the chemotherapeutic drug gemcitabine. Science.

[27] Mager LF (2020). Microbiome-derived inosine modulates response to checkpoint inhibitor immunotherapy. Science.

[28] Choi Y (2023). Immune checkpoint blockade induces gut microbiota translocation that augments extraintestinal antitumor immunity. Sci. Immunol..

[29] Helmink BA, Khan MAW, Hermann A, Gopalakrishnan V, Wargo JA (2019). The microbiome, cancer, and cancer therapy. Nat. Med..

[30] Saftien, A., Puschhof, J. & Elinav, E. Fungi and cancer. *Gut*10.1136/gutjnl-2022-327952 (2023). 10.1136/gutjnl-2022-327952 37147013

[31] Mesri EA, Feitelson MA, Munger K (2014). Human viral oncogenesis: a cancer hallmarks analysis. Cell Host Microbe.

[32] Westermann AJ, Gorski SA, Vogel J (2012). Dual RNA-seq of pathogen and host. Nat. Rev. Microbiol..

[33] Easter, Q. T. et al. Polybacterial intracellular coinfection of epithelial stem cells in periodontitis. Preprint at *bioRxiv*10.1101/2023.08.23.554343 (2023).

[34] Cossart P, Sansonetti PJ (2004). Bacterial invasion: the paradigms of enteroinvasive pathogens. Science.

[35] Weiss G, Schaible UE (2015). Macrophage defense mechanisms against intracellular bacteria. Immunol. Rev..

[36] Aliko A (2018). Impact of *Porphyromonas gingivalis* peptidylarginine deiminase on bacterial biofilm formation, epithelial cell invasion, and epithelial cell transcriptional landscape. Sci. Rep..

[37] Fattinger SA, Sellin ME, Hardt WD (2021). Salmonella effector driven invasion of the gut epithelium: breaking in and setting the house on fire. Curr. Opin. Microbiol..

[38] Mempel M (2002). Invasion of human keratinocytes by *Staphylococcus aureus* and intracellular bacterial persistence represent haemolysin-independent virulence mechanisms that are followed by features of necrotic and apoptotic keratinocyte cell death. Br. J. Dermatol..

[39] Cróinín OT, Backert S (2012). Host epithelial cell invasion by *Campylobacter jejuni*: trigger or zipper mechanism. Front. Cell Infect. Microbiol..

[40] Stelzner, K., Vollmuth, N. & Rudel, T. Intracellular lifestyle of Chlamydia trachomatis and host-pathogen interactions. *Nat. Rev. Microbiol*. 10.1038/s41579-023-00860-y (2023). 10.1038/s41579-023-00860-y 36788308

[41] Takeuchi O, Akira S (2010). Pattern recognition receptors and inflammation. Cell.

[42] Man SM, Karki R, Kanneganti TD (2017). Molecular mechanisms and functions of pyroptosis, inflammatory caspases and inflammasomes in infectious diseases. Immunol. Rev..

[43] Aguilar C, Mano M, Eulalio A (2019). MicroRNAs at the host-bacteria interface: host defense or bacterial offense. Trends Microbiol..

[44] Palmer, G. H., Bankhead, T. & Seifert, H. S. Antigenic variation in bacterial pathogens. *Microbiol. Spectr*. **4**, 10.1128/microbiolspec.VMBF-0005-2015 (2016). 10.1128/microbiolspec.VMBF-0005-2015 PMC4806564 26999387

[45] Thakur A, Mikkelsen H, Jungersen G (2019). Intracellular pathogens: host immunity and microbial persistence strategies. J. Immunol. Res..

[46] Ray K, Marteyn B, Sansonetti PJ, Tang CM (2009). Life on the inside: the intracellular lifestyle of cytosolic bacteria. Nat. Rev. Microbiol..

[47] Weddle E, Agaisse H (2018). Principles of intracellular bacterial pathogen spread from cell to cell. PLoS Pathog..

[48] Tang B (2023). MicroRNA-31 induced by *Fusobacterium nucleatum* infection promotes colorectal cancer tumorigenesis. iScience.

[49] Bullman S (2017). Analysis of Fusobacterium persistence and antibiotic response in colorectal cancer. Science.

[50] Casasanta, M. A. et al. *Fusobacterium nucleatum* host-cell binding and invasion induces IL-8 and CXCL1 secretion that drives colorectal cancer cell migration. *Sci. Signal*. **13**, 10.1126/scisignal.aba9157 (2020). 10.1126/scisignal.aba9157 PMC7454160 32694172

[51] Brennan CA, Garrett WS (2019). Fusobacterium nucleatum - symbiont, opportunist and oncobacterium. Nat. Rev. Microbiol..

[52] Ghaddar B (2022). Tumor microbiome links cellular programs and immunity in pancreatic cancer. Cancer Cell.

[53] Huang ST (2020). Intratumoral levels and prognostic significance of *Fusobacterium nucleatum* in cervical carcinoma. Aging.

[54] Yamamura K (2016). Human microbiome *Fusobacterium nucleatum* in esophageal cancer tissue is associated with prognosis. Clin. Cancer Res..

[55] Yamamura K (2019). Intratumoral *Fusobacterium nucleatum* levels predict therapeutic response to neoadjuvant chemotherapy in esophageal squamous cell carcinoma. Clin. Cancer Res..

[56] Wang X (2021). *Porphyromonas gingivalis* promotes colorectal carcinoma by activating the hematopoietic NLRP3 inflammasome. Cancer Res..

[57] Lamont RJ, Fitzsimonds ZR, Wang H, Gao S (2022). Role of *Porphyromonas gingivalis* in oral and orodigestive squamous cell carcinoma. Periodontology.

[58] Fan X (2018). Human oral microbiome and prospective risk for pancreatic cancer: a population-based nested case-control study. Gut.

[59] Okumura S (2021). Gut bacteria identified in colorectal cancer patients promote tumourigenesis via butyrate secretion. Nat. Commun..

[60] Lo CH (2022). Enrichment of *Prevotella intermedia* in human colorectal cancer and its additive effects with *Fusobacterium nucleatum* on the malignant transformation of colorectal adenomas. J. Biomed. Sci..

[61] Gursoy UK, Kononen E, Uitto VJ (2009). Prevotella intermedia ATCC 25611 targets host cell lamellipodia in epithelial cell adhesion and invasion. Oral. Microbiol. Immunol..

[62] Pushalkar S (2018). The pancreatic cancer microbiome promotes oncogenesis by induction of innate and adaptive immune suppression. Cancer Discov..

[63] Fletcher AA, Kelly MS, Eckhoff AM, Allen PJ (2023). Revisiting the intrinsic mycobiome in pancreatic cancer. Nature.

[64] Dohlman, A. B. et al. The cancer microbiome atlas: a pan-cancer comparative analysis to distinguish tissue-resident microbiota from contaminants. *Cell Host Microbe*10.1016/j.chom.2020.12.001 (2021). 10.1016/j.chom.2020.12.001 PMC7878430 33382980

[65] Fuks G (2018). Combining 16S rRNA gene variable regions enables high-resolution microbial community profiling. Microbiome.

[66] Davis NM, Proctor DM, Holmes SP, Relman DA, Callahan BJ (2018). Simple statistical identification and removal of contaminant sequences in marker-gene and metagenomics data. Microbiome.

[67] de Goffau MC (2018). Recognizing the reagent microbiome. Nat. Microbiol..

[68] Austin, G. I. et al. Contamination source modeling with SCRuB improves cancer phenotype prediction from microbiome data. *Nat. Biotechnol*. 10.1038/s41587-023-01696-w (2023). 10.1038/s41587-023-01696-w PMC10504420 36928429

[69] Westermann AJ, Vogel J (2021). Cross-species RNA-seq for deciphering host-microbe interactions. Nat. Rev. Genet..

[70] Seelbinder B (2020). Triple RNA-seq reveals synergy in a human virus-fungus co-infection model. Cell Rep..

[71] Bullman S (2023). INVADEseq to study the intratumoural microbiota at host single-cell resolution. Nat. Rev. Cancer.

[72] Guven-Maiorov E (2020). HMI-PRED: a web server for structural prediction of host-microbe interactions based on interface mimicry. J. Mol. Biol..

[73] Weiner A, Enninga J (2019). The pathogen-host interface in three dimensions: correlative FIB/SEM applications. Trends Microbiol.

[74] Strauss J (2011). Invasive potential of gut mucosa-derived Fusobacterium nucleatum positively correlates with IBD status of the host. Inflamm. Bowel Dis..

[75] Ponath F, Zhu Y, Cosi V, Vogel J (2022). Expanding the genetic toolkit helps dissect a global stress response in the early-branching species Fusobacterium nucleatum. Proc. Natl. Acad. Sci. USA.

[76] Puschhof J (2021). Intestinal organoid cocultures with microbes. Nat. Protoc..

[77] Xue C (2023). Current understanding of the intratumoral microbiome in various tumors. Cell Rep. Med..

[78] Guiney DG, Hasegawa P, Cole SP (2003). Helicobacter pylori preferentially induces interleukin 12 (IL-12) rather than IL-6 or IL-10 in human dendritic cells. Infect. Immun..

[79] Hafsi N (2004). Human dendritic cells respond to Helicobacter pylori, promoting NK cell and Th1-effector responses in vitro. J. Immunol..

[80] Bagheri N, Salimzadeh L, Shirzad H (2018). The role of T helper 1-cell response in *Helicobacter pylori*-infection. Micro. Pathog..

[81] Sigal M (2017). Stromal R-spondin orchestrates gastric epithelial stem cells and gland homeostasis. Nature.

[82] Correa P, Piazuelo MB (2012). The gastric precancerous cascade. J. Dig. Dis..

[83] Pan G (2021). Helicobacter pylori promotes gastric cancer progression by upregulating semaphorin 5A expression via ERK/MMP9 signaling. Mol. Ther. Oncolytics.

[84] Siegel RL, Miller KD, Jemal A (2020). Cancer statistics, 2020. CA Cancer J. Clin..

[85] Castellarin, M. et al. *Fusobacterium nucleatum* infection is prevalent in human colorectal carcinoma. *Genome Res.* **22**, 299–306. 10.1101/gr.126516.111 (2011). 10.1101/gr.126516.111 PMC3266037 22009989

[86] Rubinstein MR (2013). Fusobacterium nucleatum promotes colorectal carcinogenesis by modulating E-cadherin/beta-catenin signaling via its FadA adhesin. Cell Host Microbe.

[87] Liu H (2020). Fusobacterium nucleatum exacerbates colitis by damaging epithelial barriers and inducing aberrant inflammation. J. Dig. Dis..

[88] Gursoy UK, Kononen E, Uitto VJ (2008). Intracellular replication of Fusobacteria requires new actin filament formation of epithelial cells. APMIS.

[89] Dorn BR, Burks JN, Seifert KN, Progulske-Fox A (2000). Invasion of endothelial and epithelial cells by strains of *Porphyromonas gingivalis*. FEMS Microbiol Lett..

[90] Swidsinski A (1998). Association between intraepithelial *Escherichia coli* and colorectal cancer. Gastroenterology.

[91] Raisch J, Rolhion N, Dubois A, Darfeuille-Michaud A, Bringer MA (2015). Intracellular colon cancer-associated Escherichia coli promote protumoral activities of human macrophages by inducing sustained COX-2 expression. Lab. Investig..

[92] Jans, M. et al. Colibactin-induced genotoxicity and colorectal cancer exacerbation critically depends on adhesin-mediated epithelial binding. Preprint at *bioRxiv*10.1101/2023.08.16.553526 (2023).

[93] Cano CE (2012). Homotypic cell cannibalism, a cell-death process regulated by the nuclear protein 1, opposes to metastasis in pancreatic cancer. EMBO Mol. Med..

[94] Chakladar, J. et al. The pancreatic microbiome is associated with carcinogenesis and worse prognosis in males and smokers. *Cancers* **12**, 10.3390/cancers12092672 (2020). 10.3390/cancers12092672 PMC7565819 32962112

[95] Badger JL, Stins MF, Kim KS (1999). *Citrobacter freundii* invades and replicates in human brain microvascular endothelial cells. Infect. Immun..

[96] Torraca V, Holt K, Mostowy S (2020). Shigella sonnei. Trends Microbiol..

[97] Petersen AM, Krogfelt KA (2003). *Helicobacter pylori*: an invading microorganism? A review. FEMS Immunol. Med. Microbiol.

[98] Trikudanathan G, Philip A, Dasanu CA, Baker WL (2011). Association between *Helicobacter pylori* infection and pancreatic cancer. A cumulative meta-analysis. JOP.

[99] Aykut B (2019). The fungal mycobiome promotes pancreatic oncogenesis via activation of MBL. Nature.

[100] Dohlman AB (2022). A pan-cancer mycobiome analysis reveals fungal involvement in gastrointestinal and lung tumors. Cell.

[101] Urbaniak C (2016). The microbiota of breast tissue and its association with breast cancer. Appl. Environ. Microbiol..

[102] Fu A (2022). Tumor-resident intracellular microbiota promotes metastatic colonization in breast cancer. Cell.

[103] Shanmughapriya S (2012). Viral and bacterial aetiologies of epithelial ovarian cancer. Eur. J. Clin. Microbiol. Infect. Dis..

[104] Paavonen J, Turzanski Fortner R, Lehtinen M, Idahl A (2021). *Chlamydia trachomatis*, pelvic inflammatory disease, and epithelial ovarian cancer. J. Infect. Dis..

[105] Yang X (2021). *Chlamydia trachomatis* infection: their potential implication in the etiology of cervical cancer. J. Cancer.

[106] Siegl C, Rudel T (2015). Modulation of p53 during bacterial infections. Nat. Rev. Microbiol..

[107] Fischer A, Rudel T (2018). Subversion of cell-autonomous host defense by Chlamydia infection. Curr. Top. Microbiol. Immunol..

[108] Wong, L. M. et al. Comparative analysis of age- and gender-associated microbiome in lung adenocarcinoma and lung squamous cell carcinoma. *Cancers* **12**, 10.3390/cancers12061447 (2020). 10.3390/cancers12061447 PMC7352186 32498338

[109] Apostolou P (2011). Bacterial and fungal microflora in surgically removed lung cancer samples. J. Cardiothorac. Surg..

[110] Chai X (2023). Intratumor microbiome features reveal antitumor potentials of intrahepatic cholangiocarcinoma. Gut Microbes.

[111] Iida N (2021). Chronic liver disease enables gut *Enterococcus faecalis* colonization to promote liver carcinogenesis. Nat. Cancer.

[112] Nunez N (2022). The unforeseen intracellular lifestyle of *Enterococcus faecalis* in hepatocytes. Gut Microbes.

[113] Huang Y (2004). Identification of Helicobacter species in human liver samples from patients with primary hepatocellular carcinoma. J. Clin. Pathol..

[114] Kumar NG (2022). *Pseudomonas aeruginosa* can diversify after host cell invasion to establish multiple intracellular niches. mBio.

[115] Al-Hebshi NN (2017). Inflammatory bacteriome featuring *Fusobacterium nucleatum* and *Pseudomonas aeruginosa* identified in association with oral squamous cell carcinoma. Sci. Rep..

[116] Ma, J. et al. Influence of intratumor microbiome on clinical outcome and immune processes in prostate cancer. *Cancers* **12**, 10.3390/cancers12092524 (2020). 10.3390/cancers12092524 PMC7564876 32899474

[117] Kalaora S (2021). Identification of bacteria-derived HLA-bound peptides in melanoma. Nature.

[118] Konishi Y (2022). Development and evaluation of a colorectal cancer screening method using machine learning-based gut microbiota analysis. Cancer Med..

[119] Scott N, Whittle E, Jeraldo P, Chia N (2022). A systemic review of the role of enterotoxic *Bacteroides fragilis* in colorectal cancer. Neoplasia.

[120] Lamont RJ, Hajishengallis G (2015). Polymicrobial synergy and dysbiosis in inflammatory disease. Trends Mol. Med..

[121] Darveau RP, Belton CM, Reife RA, Lamont RJ (1998). Local chemokine paralysis, a novel pathogenic mechanism for *Porphyromonas gingivalis*. Infect. Immun..

[122] Saito A (2012). *Porphyromonas gingivalis* entry into gingival epithelial cells modulated by *Fusobacterium nucleatum* is dependent on lipid rafts. Micro. Pathog..

[123] Ribet D, Cossart P (2015). How bacterial pathogens colonize their hosts and invade deeper tissues. Microbes Infect..

[124] Siggins MK (2020). Extracellular bacterial lymphatic metastasis drives *Streptococcus pyogenes* systemic infection. Nat. Commun..

[125] Heikkila P, But A, Sorsa T, Haukka J (2018). Periodontitis and cancer mortality: register-based cohort study of 68,273 adults in 10-year follow-up. Int. J. Cancer.

[126] Michaud DS, Fu Z, Shi J, Chung M (2017). Periodontal disease, tooth loss, and cancer risk. Epidemiol. Rev..

[127] Han YW (2000). Interactions between periodontal bacteria and human oral epithelial cells: *Fusobacterium nucleatum* adheres to and invades epithelial cells. Infect. Immun..

[128] Gnanasekaran, J. et al. Intracellular *Porphyromonas gingivalis* promotes the tumorigenic behavior of pancreatic carcinoma cells. *Cancers* **12**, 10.3390/cancers12082331 (2020). 10.3390/cancers12082331 PMC7465784 32824786

[129] Abed J (2016). Fap2 mediates *Fusobacterium nucleatum* colorectal adenocarcinoma enrichment by binding to tumor-expressed Gal-GalNAc. Cell Host Microbe.

[130] Nakagaki H (2010). Fusobacterium nucleatum envelope protein FomA is immunogenic and binds to the salivary statherin-derived peptide. Infect. Immun..

[131] Riquelme E (2019). Tumor microbiome diversity and composition influence pancreatic cancer outcomes. Cell.

[132] Huang, J. & Huang, J. Microbial biomarkers for lung cancer: current understandings and limitations. *J. Clin. Med.* **11**, 10.3390/jcm11247298 (2022). 10.3390/jcm11247298 PMC9782454 36555915

[133] Yang M (2021). Bacteria-mediated cancer therapies: opportunities and challenges. Biomater. Sci..

[134] Chen Y, Wu FH, Wu PQ, Xing HY, Ma T (2022). The role of the tumor microbiome in tumor development and its treatment. Front. Immunol..

[135] Bertocchi A (2021). Gut vascular barrier impairment leads to intestinal bacteria dissemination and colorectal cancer metastasis to liver. Cancer Cell.

[136] Mandell GL (1973). Interaction of intraleukocytic bacteria and antibiotics. J. Clin. Investig..

[137] Vaudaux P, Waldvogel FA (1979). Gentamicin antibacterial activity in the presence of human polymorphonuclear leukocytes. Antimicrob. Agents Chemother..

[138] Gur C (2015). Binding of the Fap2 protein of *Fusobacterium nucleatum* to human inhibitory receptor TIGIT protects tumors from immune cell attack. Immunity.

[139] Yang Y (2017). *Fusobacterium nucleatum* increases proliferation of colorectal cancer cells and tumor development in mice by activating Toll-Like receptor 4 signaling to nuclear factor-kappaB, and up-regulating expression of microRNA-21. Gastroenterology.

[140] Rubinstein, M. R. et al. *Fusobacterium nucleatum* promotes colorectal cancer by inducing Wnt/beta-catenin modulator Annexin A1. *EMBO Rep.* **20**, 10.15252/embr.201847638 (2019). 10.15252/embr.201847638 PMC6446206 30833345

[141] Elsinghorst EA (1994). Measurement of invasion by gentamicin resistance. Methods Enzymol..

[142] Kim R (2019). An in vitro intestinal platform with a self-sustaining oxygen gradient to study the human gut/microbiome interface. Biofabrication.

[143] Sasaki N (2020). Development of a scalable coculture system for gut anaerobes and human colon epithelium. Gastroenterology.

[144] Puschhof J, Pleguezuelos-Manzano C, Clevers H (2021). Organoids and organs-on-chips: Insights into human gut-microbe interactions. Cell Host Microbe.

[145] Kasper SH (2020). Colorectal cancer-associated anaerobic bacteria proliferate in tumor spheroids and alter the microenvironment. Sci. Rep..

[146] Xu B, Zhou M, Qiu W, Ye J, Feng Q (2017). CCR7 mediates human breast cancer cell invasion, migration by inducing epithelial-mesenchymal transition and suppressing apoptosis through AKT pathway. Cancer Med..

[147] Jalili-Firoozinezhad S (2019). A complex human gut microbiome cultured in an anaerobic intestine-on-a-chip. Nat. Biomed. Eng..

[148] Sontheimer-Phelps A (2020). Human colon-on-a-chip enables continuous in vitro analysis of colon mucus layer accumulation and physiology. Cell Mol. Gastroenterol. Hepatol..

[149] Grassart A (2019). Bioengineered human organ-on-chip reveals intestinal microenvironment and mechanical forces impacting shigella infection. Cell Host Microbe.

[150] Avraham R (2015). Pathogen cell-to-cell variability drives heterogeneity in host immune responses. Cell.

[151] Lee P, Tan KS (2014). Fusobacterium nucleatum activates the immune response through retinoic acid-inducible gene I. J. Dent. Res..

[152] Zhang Y (2022). Fusobacterium nucleatum promotes colorectal cancer cells adhesion to endothelial cells and facilitates extravasation and metastasis by inducing ALPK1/NF-kappaB/ICAM1 axis. Gut Microbes.

[153] Yu TC (2017). *Fusobacterium nucleatum* promotes chemoresistance to colorectal cancer by modulating autophagy. Cell.

[154] Qian BZ, Pollard JW (2010). Macrophage diversity enhances tumor progression and metastasis. Cell.

[155] Kaplan CW (2005). *Fusobacterium nucleatum* apoptosis-inducing outer membrane protein. J. Dent. Res..

[156] Kaplan CW (2010). *Fusobacterium nucleatum* outer membrane proteins Fap2 and RadD induce cell death in human lymphocytes. Infect. Immun..

[157] Naghavian, R. et al. Microbial peptides activate tumour-infiltrating lymphocytes in glioblastoma. *Nature*10.1038/s41586-023-06081-w (2023). 10.1038/s41586-023-06081-w PMC10208956 37198490

[158] Yang J (2020). Guidelines and definitions for research on epithelial-mesenchymal transition. Nat. Rev. Mol. Cell Biol..

[159] Kong C (2021). *Fusobacterium nucleatum* promotes the development of colorectal cancer by activating a cytochrome P450/epoxyoctadecenoic acid axis via TLR4/Keap1/NRF2 signaling. Cancer Res..

[160] Lu X (2022). Long non-coding RNA EVADR induced by *Fusobacterium nucleatum* infection promotes colorectal cancer metastasis. Cell Rep..

[161] Ternes D (2022). The gut microbial metabolite formate exacerbates colorectal cancer progression. Nat. Metab..

[162] Chen S (2020). *Fusobacterium nucleatum* promotes colorectal cancer metastasis by modulating KRT7-AS/KRT7. Gut Microbes.

[163] Guo, S. et al. Exosomes derived from *Fusobacterium nucleatum*-infected colorectal cancer cells facilitate tumour metastasis by selectively carrying miR-1246/92b-3p/27a-3p and CXCL16. *Gut*10.1136/gutjnl-2020-321187 (2020). 10.1136/gutjnl-2020-321187 33172926

[164] Alimonti A (2004). New approaches to prevent intestinal toxicity of irinotecan-based regimens. Cancer Treat. Rev..

[165] Lehouritis P (2015). Local bacteria affect the efficacy of chemotherapeutic drugs. Sci. Rep..

[166] LaCourse KD (2022). The cancer chemotherapeutic 5-fluorouracil is a potent *Fusobacterium nucleatum* inhibitor and its activity is modified by intratumoral microbiota. Cell Rep..

[167] Serna G (2020). *Fusobacterium nucleatum* persistence and risk of recurrence after preoperative treatment in locally advanced rectal cancer. Ann. Oncol..

[168] Harish BN, Menezes GA (2015). Determination of antimicrobial resistance in Salmonella spp. Methods Mol. Biol..

[169] Buccini DF, Cardoso MH, Franco OL (2020). Antimicrobial peptides and cell-penetrating peptides for treating intracellular bacterial infections. Front. Cell Infect. Microbiol..

[170] Gomarasca, M. et al. Bacterium-derived cell-penetrating peptides deliver gentamicin to kill intracellular pathogens. *Antimicrob. Agents Chemother.* **61**, 10.1128/AAC.02545-16 (2017). 10.1128/AAC.02545-16 PMC5365713 28096156

[171] Singh R, Patil S, Singh N, Gupta S (2017). Dual functionality nanobioconjugates targeting intracellular bacteria in cancer cells with enhanced antimicrobial activity. Sci. Rep..

[172] Zheng DW (2019). Phage-guided modulation of the gut microbiota of mouse models of colorectal cancer augments their responses to chemotherapy. Nat. Biomed. Eng..

[173] Gogokhia L (2019). Expansion of bacteriophages is linked to aggravated intestinal inflammation and colitis. Cell Host Microbe.

[174] Federici S (2022). Targeted suppression of human IBD-associated gut microbiota commensals by phage consortia for treatment of intestinal inflammation. Cell.

[175] Raman V (2021). Intracellular delivery of protein drugs with an autonomously lysing bacterial system reduces tumor growth and metastases. Nat. Commun..

[176] Costa SC, Girard PA, Brehelin M, Zumbihl R (2009). The emerging human pathogen *Photorhabdus asymbiotica* is a facultative intracellular bacterium and induces apoptosis of macrophage-like cells. Infect. Immun..

[177] Wang M (2018). The roles of two type VI secretion systems in *Cronobacter sakazakii* ATCC 12868. Front. Microbiol..

[178] Kreitz J (2023). Programmable protein delivery with a bacterial contractile injection system. Nature.

[179] Avogadri F (2005). Cancer immunotherapy based on killing of Salmonella-infected tumor cells. Cancer Res..

[180] Gihawi, A. et al. Major data analysis errors invalidate cancer microbiome findings. Preprint at *bioRxiv*10.1101/2023.07.28.550993 (2023). 10.1128/mbio.01607-23 PMC10653788 37811944

[181] Kennedy KM (2023). Questioning the fetal microbiome illustrates pitfalls of low-biomass microbial studies. Nature.

[182] Queen J (2021). Comparative analysis of colon cancer-derived *Fusobacterium nucleatum* subspecies: inflammation and colon tumorigenesis in murine models. mBio.

[183] Tomkovich S (2017). Locoregional effects of microbiota in a preclinical model of colon carcinogenesis. Cancer Res..

[184] Chin VK (2020). Mycobiome in the gut: a multiperspective review. Mediators Inflamm..

[185] Ogunrinola GA, Oyewale JO, Oshamika OO, Olasehinde GI (2020). The human microbiome and its impacts on health. Int. J. Microbiol..

[186] Krump NA, You J (2018). Molecular mechanisms of viral oncogenesis in humans. Nat. Rev. Microbiol..

[187] Dohlman AB (2021). The cancer microbiome atlas: a pan-cancer comparative analysis to distinguish tissue-resident microbiota from contaminants. Cell Host Microbe.

[188] Narunsky-Haziza L (2022). Pan-cancer analyses reveal cancer-type-specific fungal ecologies and bacteriome interactions. Cell.

